# Human Marfan and Marfan-like Syndrome associated mutations lead to altered trafficking of the Type II TGFβ receptor in *Caenorhabditis elegans*

**DOI:** 10.1371/journal.pone.0216628

**Published:** 2019-05-09

**Authors:** Jing Lin, Mehul Vora, Nanci S. Kane, Ryan J. Gleason, Richard W. Padgett

**Affiliations:** 1 Waksman Institute, Department of Molecular Biology and Biochemistry, Rutgers University, Piscataway, New Jersey, United States of America; 2 Department of Biology, Johns Hopkins University, Baltimore, Maryland, United States of America; INSERM U869, FRANCE

## Abstract

The transforming growth factor-β (TGFβ) family plays an important role in many developmental processes and when mutated often contributes to various diseases. Marfan syndrome is a genetic disease with an occurrence of approximately 1 in 5,000. The disease is caused by mutations in fibrillin, which lead to an increase in TGFβ ligand activity, resulting in abnormalities of connective tissues which can be life-threatening. Mutations in other components of TGFβ signaling (receptors, Smads, Schnurri) lead to similar diseases with attenuated phenotypes relative to Marfan syndrome. In particular, mutations in TGFβ receptors, most of which are clustered at the C-terminal end, result in Marfan-like (MFS-like) syndromes. Even though it was assumed that many of these receptor mutations would reduce or eliminate signaling, in many cases signaling is active. From our previous studies on receptor trafficking in *C*. *elegans*, we noticed that many of these receptor mutations that lead to Marfan-like syndromes overlap with mutations that cause mis-trafficking of the receptor, suggesting a link between Marfan-like syndromes and TGFβ receptor trafficking. To test this hypothesis, we introduced three of these key MFS and MFS-like mutations into the *C*. *elegans* TGFβ receptor and asked if receptor trafficking is altered. We find that in every case studied, mutated receptors mislocalize to the apical surface rather than basolateral surface of the polarized intestinal cells. Further, we find that these mutations result in longer animals, a phenotype due to over-stimulation of the nematode TGFβ pathway and, importantly, indicating that function of the receptor is not abrogated in these mutants. Our nematode models of Marfan syndrome suggest that MFS and MFS-like mutations in the type II receptor lead to mis-trafficking of the receptor and possibly provides an explanation for the disease, a phenomenon which might also occur in some cancers that possess the same mutations within the type II receptor (e.g. colon cancer).

## Introduction

Marfan syndrome is an autosomal dominant genetic disorder of connective tissue that affects the ocular, skeletal, cardiovascular and pulmonary systems. Major cardiovascular manifestations, including aortic root dilatation, dissection and rupture, pulmonary artery dilatation, mitral and aortic valve insufficiency, often lead to death in early adult life. The pleiotropic manifestations of Marfan syndrome can be directly attributed to germline mutations in fibrillins. Several studies have provided convincing evidence that fibrillin mutations are associated with ineffective sequestration of TGFβ ligand in the matrix, which is believed to lead to excessive levels of bioactive TGFβ in the tissue microenvironment [1, 2]

Given the involvement of increased TGFβ ligand in Marfan syndrome, it is not surprising to find that mutations in other components of the TGFβ pathway can result in related disorders, collectively termed MFS-like syndromes [3–5], including Marfan syndrome 2 (MFS2), Loeys-Dietz syndrome (LDS), Ehlers-Danlos syndrome (LDS-2), Thoracic Aortic Aneurysms and Dissections (TAAD) and Shprintzen-Goldberg syndrome (SGS). The common thread of each of these disorders is that they show milder manifestations of many of the phenotypes seen in Marfan syndrome.

The TGFβ family includes a large family of secreted, soluble proteins that act as growth factors; they are dimeric multifunctional regulators playing important roles in various embryonic and developmental processes from invertebrates to mammals [6]. Based on their roles in signal transduction, the TGFβ receptors are subdivided into three classes, type I, II and III [7]. The transmembrane type I and type II receptors are the major signaling receptors interacting with ligands [7].

Both type I and type II receptors are related to each other and are heterodimeric transmembrane kinases most closely related to serine/threonine kinases [8]. There are cysteine-rich regions for ligand binding in the extracellular domain of the N-terminus in both receptors [9]. The kinase domain, an essential factor in TGFβ receptor function, occupies most of the cytoplasmic domain in the C-terminus [9]. Binding of ligand to the extracellular domain of the TGFβ type II receptor leads to a set of conformational changes in the intracellular domain of the receptor, which allows the phosphorylation and subsequent activation of the type I receptor [8]. Receptor activation triggers downstream TGFβ signaling. SMAD proteins are the primary downstream substrate for the activated type I receptor kinase in the canonical pathway [6]. R-SMAD is activated through phosphorylation by an activated type I receptor and translocates into the nucleus to regulate specific gene expression with the assistance of co-SMAD.

**Fig 1:**
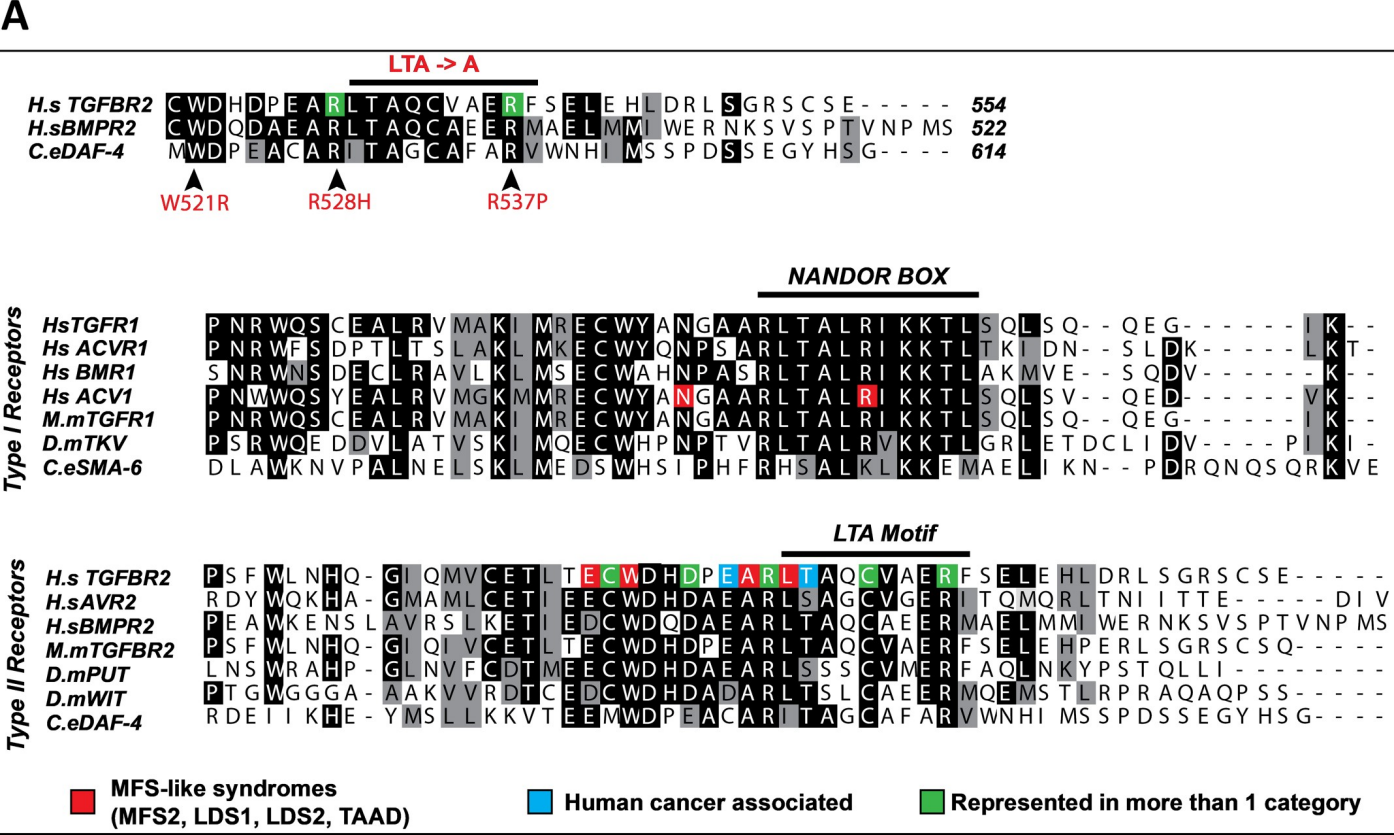
**A.** Top Panel: Amino acid alignment of the LTA motifs of the human type II TGFβ and BMP receptors with the type II receptor of *C*. *elegans*. Arrows indicate the three mutations we have examined in detail in this study and the region where the LTA motif was substituted with alanines. Middle and Lower Panels: Amino acid alignments of the various type I and type II TGFβ receptors highlight the sequence conservation at the NANDOR box and LTA motif respectively. Black indicates identity, grey indicates conserved changes. For both panels, the various colored boxes identify the residues found mutated in either MFS-like patients, cancers, or in multiple categories (red, blue, orange and green respectively). As observed, all mutations exist as missense mutations in the type II TGFβ receptor.

Most of these MFS-like disorders can be attributed to heterozygous germline mutations of either the type II (*TGFΒR2*) or type I (*TGFΒR1*) TGFβ receptor genes (Fig 1). Less commonly found are mutations in other signaling components of TGFβ, such as Smad3 [10, 11], TGFΒ2 [12], TGFΒ3 [13] and the repressor SKI [14]. Interestingly, in almost every case described to date, the mutation is a missense mutation suggesting production of a full-length protein.

Substantial experimental evidence suggests that constitutive activation of the TGFβ signaling pathway is at least partly responsible for the vascular abnormalities seen in classic Marfan syndrome [1, 2]. However, the story with MFS-like syndromes is much more complicated, with reports of increased or decreased TGFβ signaling, depending on what was assayed and which signaling component was examined [3, 15]. In samples from Loeys-Dietz patients (LDS), aortic tissue showed elevated pSmad2/3 levels, but the mutated receptors showed little intrinsic kinase activity [16], highlighting the paradox. In summary, most researchers agree that the aortic root wall (the major focus of defects in the MFS-like diseases) shows increased TGFβ signaling in human patients and in mouse models of MFS-like syndromes [1] with a reduction of kinase signaling potential from the mutated receptors. How the heterozygous loss-of-function of the receptors contributes to increased downstream signaling remains paradoxical [1, 2, 12].

Progress has been made in understanding how these mutations found in MFS-like syndromes affect the function of the TGFβ receptors, but a clear understanding is still missing [1–3, 12, 17–21]. For most MFS-like receptor mutations that have been examined, no receptor kinase activity has been detected *in vitro*, and no pSmad2 stimulation was observed when exposed to ligand [3]. In these MFS-like mutants, internalization of the receptors was mildly diminished in some mutants, but not significantly distinguishable from wild type [3], indicating that synthesis and secretion of the receptors were normal.

We noticed that mutated receptor sequences in MFS-like syndromes overlap extensively with sequences at the C-terminal tails of the type I (NANDOR Box: non-activating and non-down-regulating) and type II (LTA motif comprising of leucine, threonine and alanine) that are needed for receptor trafficking (internalization as well as sub-cellular localization of both receptors) [22, 23]. In addition, Zhou *et al*. (2004), found a nearby tryptophan located a few amino acids upstream of the NANDOR box that is necessary for the baso-lateral delivery of the type I receptor, further supporting a role for this region in intracellular receptor trafficking [24]. An alignment of the C-terminal tails for type I and II receptors from nematodes to mammals highlights the related NANDOR box and LTA motif, special amino acids and endocytic regions (Fig 1A). Most of the amino acids in the NANDOR box or the LTA motif are highly conserved across species, and the endocytic regions in both the type I and type II receptors have a significant overlap with each other.

Based on our observation that trafficking motifs in the type I and type II receptors overlap with MFS, MFS-like and some cancer mutations, we hypothesized that some of these phenotypes could be due to mis-trafficking of the TGFβ receptors. Therefore, we sought to test this hypothesis using the model organism *C*. *elegans* where trafficking studies on TGFβ are well-established.

## Results

### Receptor mutations define a critical domain for disease and trafficking

Patients afflicted with MFS-like diseases generally contain mutations in genes that are components of or regulate the TGFβ signaling pathway [14, 25]. A large proportion of these mutations are present within the receptors. Three heterozygous missense mutations in the TGFβ type II receptors of MFS-like patients were chosen for detailed examination (Fig 1): W521R [4], R528H (which was identified in 6 out of 10 families with MFS-like syndrome [26]), and R537P [3]. The corresponding positions in the *C*. *elegans* type II receptor, DAF-4, are W580R, R587H and R596P respectively.

Additionally, different missense mutations in TGFβ receptors have been identified in human cancer cell lines or tumor specimens [26–36]. As in the case of the MFS-like mutations, many of the cancer-associated mutations in the *TGFΒR2* gene cluster in the same C-terminal domain [37–39] (Fig 1). These mutations occur in the *TGFΒR2* gene (> 40 mutations) but also in the *TGFΒR1* gene (4 mutations) (Fig 1). The MFS-like R528H mutation is also present in type II receptors in colon carcinoma cell lines [27]. Likewise, the mutation R537P is mutated in both MFS-like patients and some cancers [3, 40]. The overlap between the mutations present in MFS-like and TGFβ-linked cancers suggests that some of these cancers might also be caused by mis-trafficking of the receptors.

Further mutational analysis of TGFβ receptors shows this C-terminus to be important for signaling. A human mutation associated with brachydactyly resides in the BMP type I receptor (related to the TGFβ family of receptors) in this same C-terminal domain [41], highlighting that both TGFβ and BMP receptors are susceptible to mutation in this conserved domain (Fig 1). The mutations described above reside primarily in a motif that overlaps with the LTA motif **(**residues 529–538 underlined) that disrupted the normal trafficking of the receptors (Fig 1).

We sought clues to how this C-terminal domain might be involved in trafficking by examining its 3D structure. Using the available structure of the type II TGFβ receptor (PDBID: 5e8V), we observe that the LTA motif of the kinase domain is exposed to the exterior where interactions with other proteins, particularly trafficking regulators might be expected to occur (S1A Fig). To investigate how the MFS-like mutations might interfere with the structure and function of the human type II TGFβ receptor, we modeled them in Pymol [42] to better understand the relationship between various residues in the LTA motif and the larger structure of the protein (S1B Fig). In the native state of the wild-type type II TGFβ receptor, residue W521 contributes to a hydrophobic core that stabilizes the pairing of helices 8 and 12. This hydrophobic core is composed of residues W521, L452, P498, W455, I500 and L512. The mutation W521R can cause a destabilization of the pairing between helices 8 and 12, with a rearrangement of the other side-chains in the core to compensate for the loss of the hydrophobic moiety of the tryptophan. R528 and R537, in helices 13 and 14, respectively, are involved in long range electrostatic interactions with oppositely charged residues. R528 forms a salt bridge with E428 in helix 7, and R537 forms a salt bridge with E519 in helix 12. These interactions contribute to stability of the heli​cal​ bundle in the C-terminal domain of the type II TGFβ receptor. The mutation R528H may affect the pairing of helix 13 with helix 7, but due to the limited extent of helix 13 (4 residues), the mutation ​may have little importance for the stability of the secondary and tertiary structure of the domain or interactions with partner proteins, as the two residues are relatively poorly exposed to the solvent. The mutation R527P, instead, may be important both for the stability of helix 14, since proline is a helix-breaker, and for the pairing of the solvent-exposed helix 14 with helix 12. Moreover, R537 is on the protein surface, and may belong to a binding site for partner proteins. Given the high degree of conservation within the LTA motif as well as the kinase domains of the human and nematode we expect similar structural changes in the worm receptor.

In summary, the structural modeling suggests that the mutations found in the MFS-like patients would disrupt the structure of an exposed surface domain of the type II TGFβ receptor, perhaps altering its interaction with cytoplasmic trafficking and/or regulatory proteins or the function of the receptor.

### Type II TGFβ receptors bearing MFS-like mutations are functional

How each of the mutations affects receptor activity and downstream signaling has been controversial [1]. Most of these studies are *in vitro*, where the cellular context of TGFβ is removed, particularly those elements involved in receptor trafficking. Whether disease-associated mutations within the type II TGFβ receptors alter signaling strength and output is not clear; some studies have identified that mutations within the LTA motif disrupt the kinase activity while others have shown that Smad phosphorylation is unaffected [1, 2]. It is important to note that all the mutations described above are missense mutations and should produce a full-length product. Most of these residues do not alter Smad2/3 *in vitro* phosphorylation [23]. Of note, two residues that resulted in a loss of Smad phosphorylation were alanine substitutions rather than naturally occurring mutations like those found in MFS-like [23], which may explain why these two substitutions behaved differently in these assays. Importantly, Smad phosphorylation was comparable to wild-type levels for mutations at all other residues in the domain [23]. It is important to note that although Smad2/3 phosphorylation levels have been studied for this region, there are no studies that assay signaling strength for alanine substitutions within this region or in the context of a disease-mutation. To identify whether the presence of MFS-like mutations affects function of the receptors *in vivo*, we took advantage of the fact that TGFβ signaling governs body length in the worm [43–46]. Loss of TGFβ signaling (*e*.*g*. mutations in the type II receptor) leads to a smaller body length, and body length can be rescued (or enhanced) by transgenic overexpression of signaling components specifically in the hypodermis.

**Fig 2:**
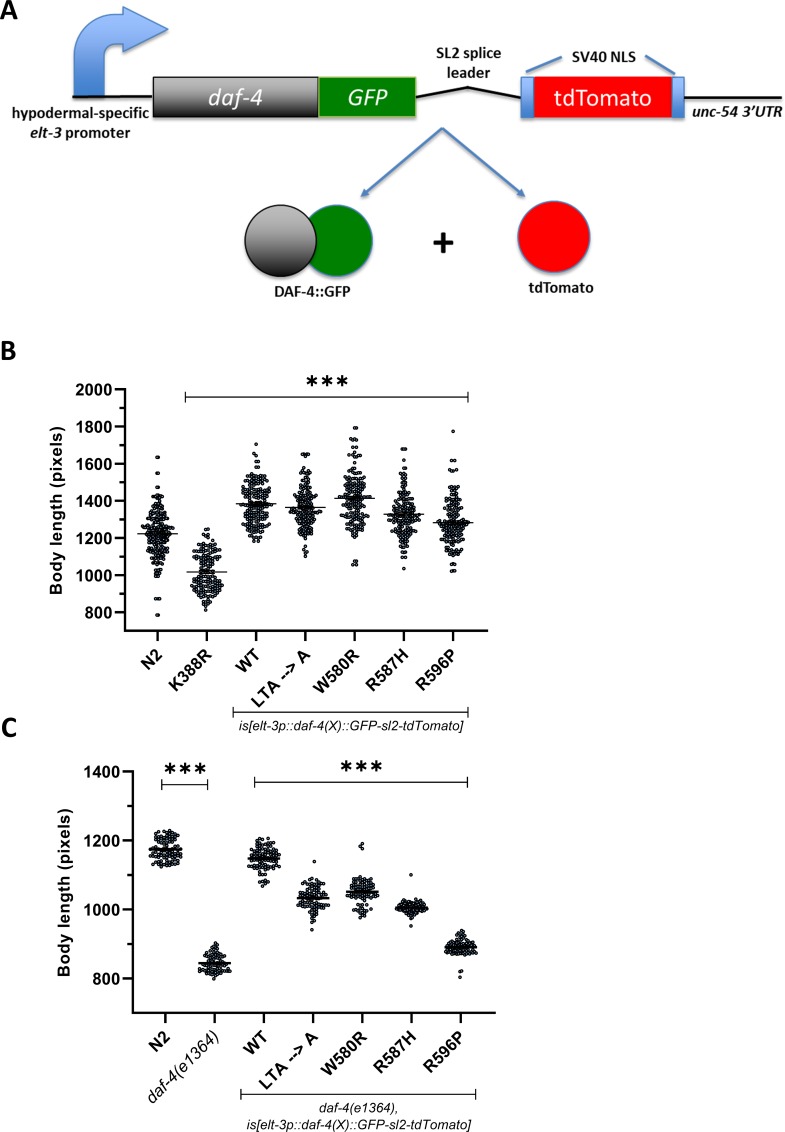
**A.** Schematic overview of the transgenic construct to determine the functionality of the type II TGFβ receptors bearing MFS-like mutations. The SL2 splice leader makes it possible for the one-to-one expression of the DAF-4 and NLS-tdTomato-NLS cassette. This allows quantification of the levels of transcription from the hypodermal specific promoter *elt-3*. **B., C.** Hypodermal specific transgenic expression of type II TGFβ receptors bearing either the LTA → A substitutions or the MFS-like mutations are capable of increasing body length in wild-type animals (B), or partially rescuing the body size of *daf-4(e1364)* mutant animals (C). K338R is a substitution mutation that generates a kinase-dead receptor. Graph shows the mean body lengths values +/- S.E.M. Statistical comparisons were performed using a One-way ANOVA with Dunnett’s correction for multiple comparisons against wild-type or *daf-4(e1364)* strains. These data suggest that MFS-like mutations do not abrogate receptor function. A minimum of 30 animals were measured for each genotype.

With the advent of CRISPR/Cas9 technology to seamlessly modify endogenous loci [47], we sought to modify the endogenous *daf-4* locus. We received a C-terminal GFP-tagged *daf-4* strain from Dr. Jun Liu at Cornell University (a gift). Unfortunately, the fluorescent signal from the GFP-tagged protein was undetectable or very weak so that it would not be useful for imaging purposes, a problem that can arise when transcription levels of a gene are low [48]. Thus, in order to address the functionality and the localization patterns of the Type II TGFβ receptor, we chose to create transgenic animals that bear integrated low-copy transgenes [49] of wild-type or MFS-mutant receptors. The transgenic cassette consisted an operon of the *daf-*4 gene and an *NLS-tdTomato-NLS* separated by a SL-2 trans-splice leader sequence [50] driven under the control of the hypodermal specific *elt-*3 promoter (Fig 2A). The tdTomato acts as an internal control to facilitate normalization for transgene number across different strains. Transgenic animals were generated by microparticle bombardment of the constructs using established methods [49]. After selection of lines and outcrossing to our lab wild-type strain, we show that transgene expression levels are comparable across all transgenic lines as determined by tdTomato quantification (S2 Fig) where there was no statistical difference amongst the strains; thus any changes in body size amongst strains is an intrinsic function of the MFS-mutation.

We first show that overexpression of the wild-type *daf-4* led to an increase in the body length of the wild-type animal re-iterating that enhanced signaling leads to a longer body length. Expression of a kinase-dead version of the receptor [40] led to a smaller animal (p < 0.0001, Fig 2B, N = 3, all individual values across replicates combined, n ≥ 30 animals per trial). Thus, the presence of a ‘poison’ receptor in a pool of wild-type of *daf-4* receptors leads to a dominant negative phenotype of a small body size, perhaps through competition with the wild-type receptor in the heterotetrameric signaling complex of type I and II receptors. Given these two results, we expected one of three different outcomes for the MFS-mutant receptors: 1) If we observe no change in the body length of the transgenic animal, the receptor may be non-functional, 2) If we observe a smaller body size, it could indicate that the mutant receptor behaves in a dominant negative manner with the wild-type receptor, and 3) If we observe a longer body size the receptor may be active and functional.

We first showed that mutating the entire LTA motif with alanines does not disrupt receptor function, as evidenced by the longer body length which requires active TGFβ signaling. Importantly, we have shown that the W580R and R587H mutants result in functional receptors (Fig 2B) even though their expressed levels are lower than wild-type receptors.

However, the presence of the wild-type (endogenous) copy of the *daf-4* gene may influence the activity of the transgenic mutant receptors. Hence, we crossed each of our transgenic lines into the *daf-4(e1364)* mutant [51], a null mutant with a small body size phenotype. Hypodermal expression of wild-type copies of the transgenic *daf-4* gene in this mutant fully rescues the small body length of the *daf-4(e1364)* mutant (Fig 2C, N = 3, all individual values across replicates combined, n ≥ 30 animals per trial). We observe that all MFS-mutant transgenes rescue the small body size of the *daf-4(e1364)* mutant, albeit to different levels. This suggests that although all the MFS-mutant receptors are functional, there exists variation in their signaling strength. Taken together, these data strongly suggest that MFS-mutations do not fully attenuate type II TGFβ receptor activity as assayed *in vivo* in a whole organism context.

### LTA motif and disease-associated MFS mutations lead to reduced levels of the type II TGFβ receptor

**Fig 3:**
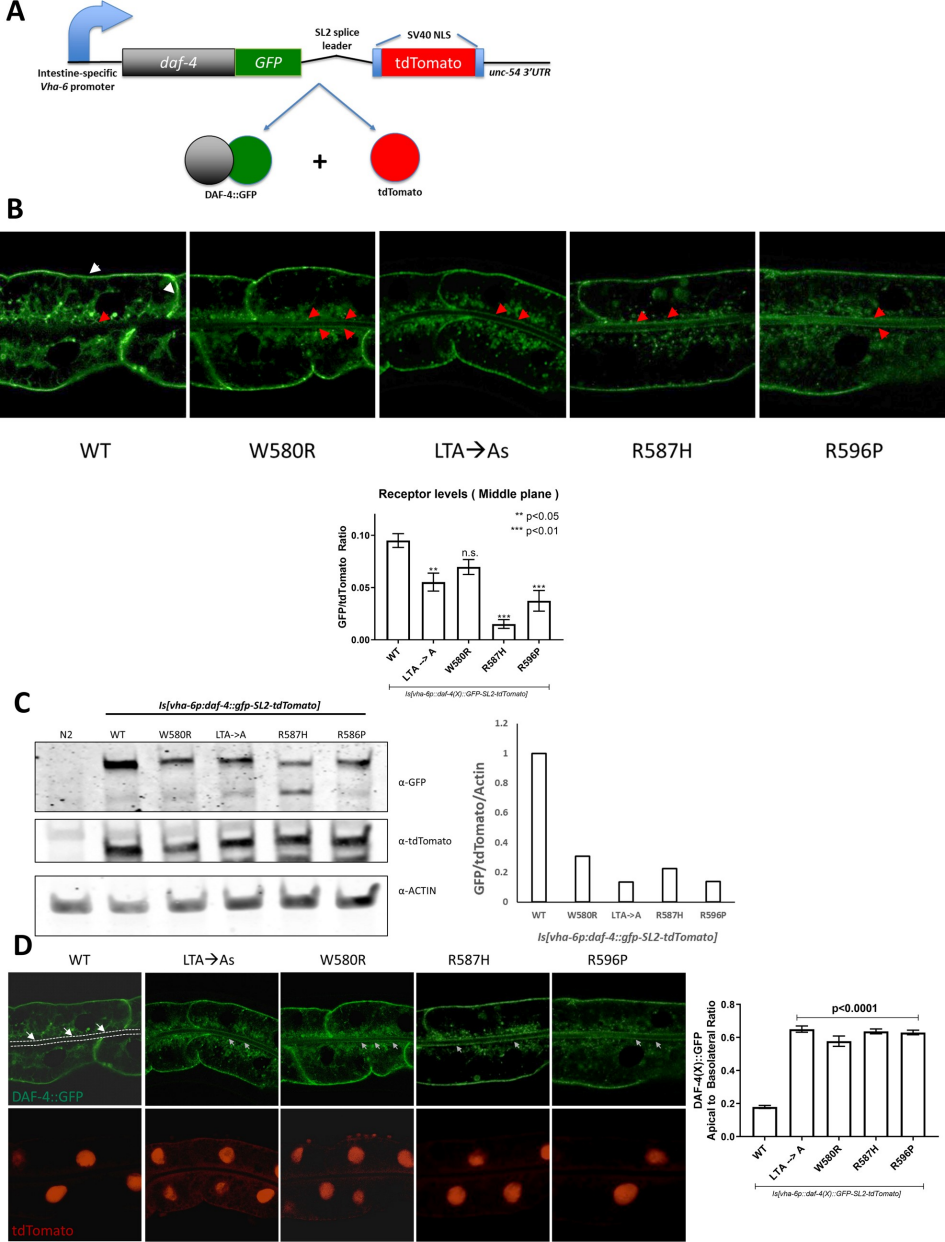
**A.** Schematic overview of the transgene construct to study cellular levels and localization patterns of type II TGFβ receptors bearing MFS-like mutations. The SL2 splice leader makes it possible for the one-to-one expression of the DAF-4::GFP and NLS-tdTomato-NLS cassette. This allows precise quantification of the levels of the GFP-tagged DAF-4 receptor. The coding sequences are expressed by the intestine specific *Vha-6* promoter. **B., C.** The LTA → A and MFS-like mutations lead to significantly decreased DAF-4 receptors intracellularly as determined by confocal microscopy (B) and western blot (C). At least six animals were imaged for confocal imaging with three biological replicates per genotype, and quantification of fluorescence intensity was carried out for intracellular abundance. All images have been exposed at the same settings. Graphs indicate mean intensity +/- S.E.M. Statistical comparisons were performed using a One-way ANOVA with Dunnett’s correction for multiple comparisons against the wild-type strain. Western blot shown is a representative blot from at least three different biological replicates. For Fig 3B, red arrows indicate apical surfaces while white arrows indicate basolateral surfaces. **D.** Overexposure of the confocal images reveals that the low levels of mutant receptor present intracellularly are mis-trafficked to the apical surface of the intestine (white arrows) as compared to the basolateral surface. Dotted line in WT panel indicates the apical cellular limit. Quantification of the ratio of the apical to basolateral surface is shown in the attached graph. These data indicate that the LTA motif is required for the proper cellular distribution of the DAF-4 receptor and that the MFS-like mutations may disrupt interactions that could affect cellular distribution. Graphs indicate mean intensity +/- S.E.M. Statistical comparisons were performed using a One-way ANOVA with Dunnett’s correction for multiple comparisons against the wild-type strain.

To visualize levels and localization of the mutant receptors *in vivo*, we expressed them in the *C*. *elegans* intestine. The polarized epithelial cells of the intestine are a well characterized system for the study of the receptor trafficking as well as a tissue in which TGFβ signaling is required and active [52–54]. To this end, we developed a vector that contains an operon consisting of a *daf-4* receptor with a GFP-tag at the C-terminus (wild-type and point mutants) and an *NLS-tdTomato-NLS* separated by a SL-2 trans-splice leader sequence [50], expressed from the intestinal specific *vha-6* promoter. Upon transcription and splicing, the result is a single GFP-tagged receptor and a single tdTomato molecule containing dual nuclear localization signals (Fig 3A). The GFP-tagged receptors allow for easy visualization of trafficking, while the tdTomato acts as an internal control to detect subtle changes in the receptor expression levels as well as normalization for transgene number across different strains. This construct was stably integrated at a low-copy number into the worm genome using microparticle bombardment [49].

Confocal imaging (horizontal section of the worm intestine; white arrow represents basolateral surfaces while red arrow indicates apical or lumenal surfaces, Fig 3B) and western blot analyses revealed that changes in the LTA motif, either substituting the motif with alanines or with MFS-like mutations, resulted in significantly decreased levels of receptors (Fig 3B and 3C). To rule out the possibility that the reduced levels of mutant type II receptor were due to any aberrant synthesis and/or transport to the plasma membrane, we examined the accumulation of tagged receptors at the plasma membrane by knocking down *dpy-23*. *dpy-23* is the μ-2 adaptin member of the AP2 complex and is required for the clathrin-dependent endocytosis of various transmembrane receptors, including BMP/TGFβ receptors [52, 55]. Loss of *dpy-23* by RNAi leads to the suppression of clathrin-mediated endocytosis, resulting in accumulation of the type I receptor SMA-6 at the plasma membrane (loss of internalization) and inhibition of BMP signaling [52], and we used this particular assay as our control to determine efficacy of the RNAi knockdown of *dpy-23*. As previously observed, knockdown of *dpy-23* did not significantly impact the accumulation or levels of either wild-type or mutant DAF-4 receptors, suggesting that biosynthesis and transport to the plasma membrane are likely not impaired by these MFS mutations (S3 Fig).

Lysosomes are the degradative endpoints of most cell surface receptors [56–58]. Given that MFS-mutant receptors are present at lower levels within the cell, it is possible that the MFS-mutant receptors exhibit altered cellular kinetics and are degraded within the lysosome at increased rates. To examine this possibility, we performed a knockdown of *cup-5*, a gene necessary for lysosome function in *C*. *elegans* whose knockdown leads to dysfunctional lysosomes. Proteins that are normally degraded are expected to accumulate intracellularly in a *cup-5* mutant or knockdown background. We have previously shown that the loss of the retromer subunit, *vps-35*, leads to the premature degradation of the type I receptor SMA-6 within the lysosome which can be reversed by RNAi mediated knockdown of *cup-5 [52]*. We hypothesized that if the mutant DAF-4 receptors were being degraded in the lysosome, we would observe an accumulation of the GFP-tagged receptor if the lysosome function was blocked as these receptors would no longer be degraded within the lysosome. However, there was no change in the receptor levels upon *cup-5* RNAi, suggesting that the MFS-like mutations did not lead to an increased rate of degradation within the lysosome (S4 Fig).

Taken together, the data from the *dpy-23* and *cup-5 RNAi* experiments suggest that the lowered levels of the MFS-mutant type II receptor are not a result of defective synthesis, transport to the plasma membrane or degradation of the receptor in the lysosomes.

### Subcellular localization patterns are altered by MFS-mutations

Although total receptor levels were reduced, we observed that the mutant receptors remaining within the cell displayed a markedly different localization pattern compared to the wild-type receptors (Fig 3D). Images have been overexposed to easily identify localization changes; quantification is based on unmodified exposures. As we have previously observed [52], the wild-type TGFβ type II receptors are located along the basolateral membranes and in a net-like distribution with little to no localization along the apical cell boundary (Fig 3D, white arrows represent apical surface, dotted line is the cell boundary). Alteration of the LTA motif or introduction of the MFS-mutations leads to the loss of the net-like distribution and a significantly increased localization to the apical surface (Fig 3D, white arrows). Structure-function of truncation mutants and alanine substitutions in the LTA motif have been shown to be important for localization to the apical and basolateral surfaces in polarized MDCK cell lines [23]. Our *C*. *elegans* models of MFS-like mutations found in patients, rather than alanine substitutions, result in altered trafficking of the type II TGFβ receptor from the basolateral surfaces to the apical surface of the cell.

### Mutant TGFβ type II receptors alter the level and trafficking of the type I receptor

Signaling requires a heterotetrameric complex of type II and type I receptors; the receptors must overlap spatially for the complexes to form upon ligand binding to the type II receptor. Upon internalization, the two receptors separate at some point and recycle back to the surface via distinct mechanisms, with the type I receptor recycling via the retromer and the type II receptor utilizing an *arf-6* mediated recycling pathway [52].

**Fig 4:**
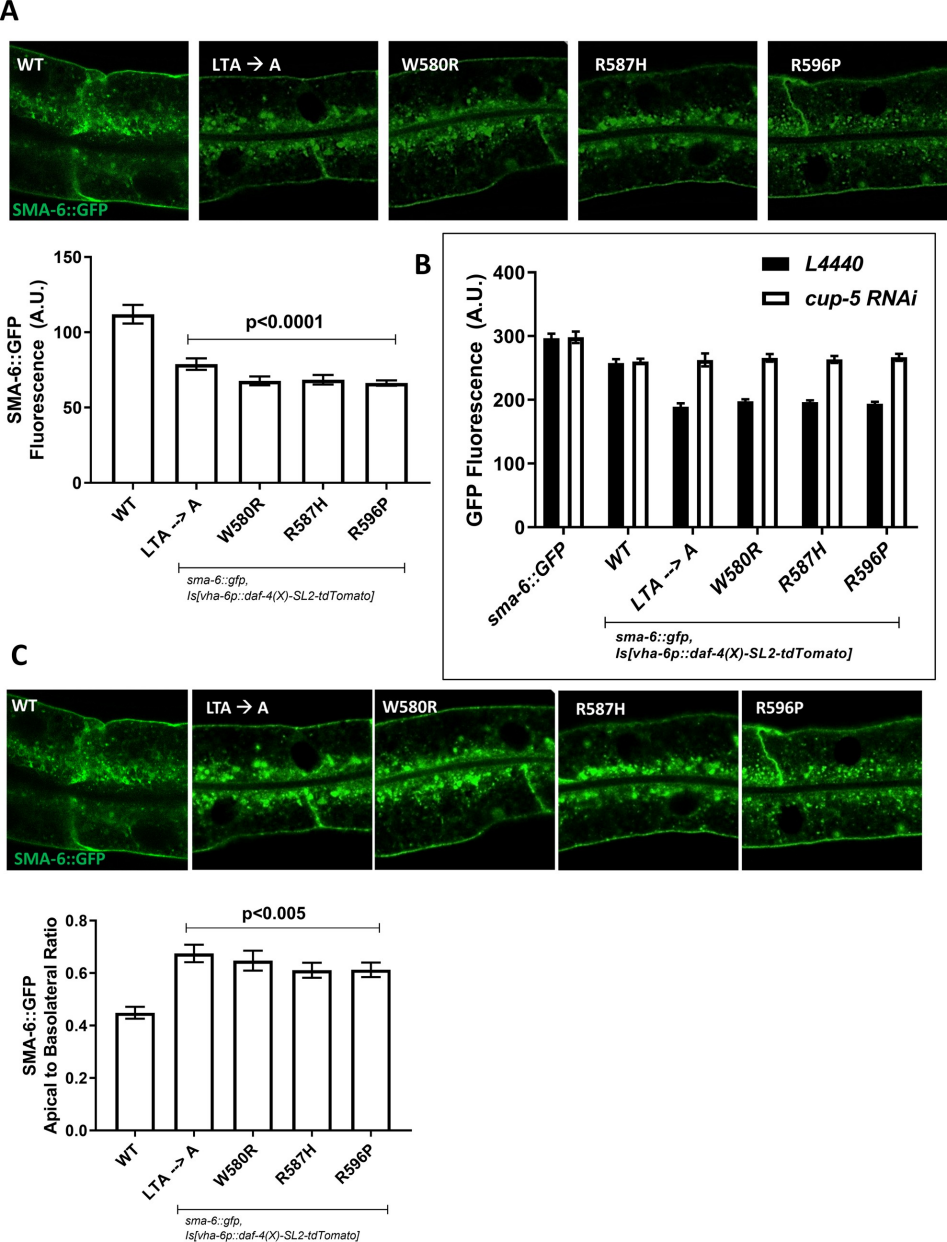
**A.** Type I TGFβ receptor levels are significantly (p < 0.0001) lowered in the presence of MFS-like mutant type II TGFβ receptors as determined by confocal imaging. A GFP-tagged type I receptor SMA-6 was co-expressed in the intestine with an untagged wild-type or MFS-like mutant type II receptor. **B.** The type I receptor, SMA-6, degraded in the presence of the MFS-like type II receptor was restored by inhibition of the lysosome function by knockdown of *cup-5* RNAi. **C.** Overexposure of the confocal images reveals that the presence of mutant type II receptors significantly (p < 0.005) altered the intracellular distribution of the type I receptors from the basolateral surfaces to the apical/sub-apical surfaces of the intestine.

We have shown that the presence of MFS-like mutations in the type II receptor leads to the shift in localization from the basolateral surface to the apical surface of the intestine (Fig 3D). The increase in body size in the wild-type background and rescue of the small body phenotype of the *daf-4(e1364*) mutant with the MFS-like mutant receptors, strongly suggests that activity is not diminished with these mutations (Fig 2). Given the new localization pattern of the mutant DAF-4 receptors and that SMA-6 would be required at the same locations, we asked what the effect of the MFS-like mutations were on the cell biology of the type I receptor, SMA-6. We tested the levels and localization of a functional *sma-6*::*gfp* transgene within the intestine in the presence of either wild-type or MFS-like mutant *daf-4* receptors (untagged but expressing a tdTomato through an SL2 like in Fig 2A.). We first show that the total levels of transgene expression are the same across strains as assayed by tdTomato levels (S5 Fig) indicating that levels of *daf-4* transcript are similar across all strains. We observe a significant decrease in overall levels of the type I receptor within the cell when co-expressed with the mutant DAF-4 receptors (Fig 4A). The type I BMP receptor is known to recycle through a retromer-mediated mechanism, and a loss of this retromer-receptor interaction leads to degradation of the type I receptor in the lysosome. To identify whether the type I receptor was indeed being degraded in the lysosome, we hypothesized that perturbation of lysosome function would lead to a recovery of GFP-tagged receptors that are normally degraded within the lysosome. An RNAi of *cup-5* led to the restoration of the SMA-6:GFP signal indicating that the presence of MFS-mutant type II receptors leads to abnormal degradation of the type I receptors within lysosomes. (Fig 4B).

In the presence of the wild-type DAF-4 receptor, SMA-6::GFP localizes primarily to the basolateral surfaces of the intestinal cells (Fig 4C), as has previously been shown [52]. In stark contrast, the presence of the mutant DAF-4 receptors leads to a change in localization from the basolateral surface to the apical surface, similar to what we observe for the mutant DAF-4 receptors themselves i.e mislocalization proximal to the apical surfaces (Fig 3). The change in localization of SMA-6 in the presence of the mutant DAF-4 receptors suggests that the receptors are dependent on each other for trafficking. Whether this dependency occurs after internalization and separation during recycling or soon after biosynthesis and transport to the plasma membrane needs to be further examined.

## Discussion

### MFS-like mutations do not disrupt function of the receptor

A hallmark of Marfan’s and MFS-like syndromes is an increase in TGFβ signaling. The prevalence of MFS-like mutations at the LTA motif of type II receptors, which lies within the kinase domain, can potentially lead to defective kinase activity [1, 2, 12]. *In vitro* structure-function assays determining signal output through levels of pSMAD or reporter genes are controversial–some studies observed lowered pSMAD activation while others observed no change in pSMAD levels. In fibroblasts from a TGFβ kinase-deficient mouse, increased signaling was unexpectedly seen in assays of pSmad2 [59]. Others have shown that mutant TGFβ receptors maintained kinase activity and that phosphorylated R-Smads were detected [41]. In MFS-like patients, it has been demonstrated that specific missense mutations screened within TGFβ receptors caused reduced acute responsiveness to ligand, but long-term signal transduction was not disrupted [19]. More recently, in another mouse model containing an MFS-like mutation engineered into the TGFβ type II receptor, impaired receptor signaling was observed in osteoblasts, but cultured osteoblasts had increased differentiation markers compared to wild type [59]. The authors attribute these results to possible deficiencies in modeling the disease *in vitro*.

In our study, we created an *in vivo* model of MFS-like syndromes by introducing human MFS-like mutations, and not alanine substitutions, into the *C*. *elegans* type II TGFβ receptor and studying it in a whole animal context. Our models clearly show that the type II receptors bearing MFS-like mutations are functional as determined by a body size assay. The increase in body size in a wild-type background resembles the dominant nature of these mutations found in MFS-like patients.

### MFS-like mutations alter type II receptor trafficking and, indirectly, type I receptor trafficking

The LTA-domain is required for sorting of the type II TGFβ receptor to the basolateral surface and acts in a dominant manner; e.g. when the domain is transferred to an apically localized influenza haemagglutinin (HA) antigen, it directs a dominant mis-localization to the basolateral surface [23]. While several studies have examined important aspects of receptor function in MFS-like diseases, the intracellular trafficking of the mutant receptors had not been examined, particularly in the context of a whole animal. Given the coincidence of these disease mutations with an experimentally identified domain that is required for receptor trafficking, examination of intracellular trafficking was warranted, and likely contributes to the phenotype.

Our *in vivo* assays found that MFS-like associated mutations in or around the defined LTA motif in the type II receptor affect the trafficking of *both* TGFβ receptors, although the effect on the type I receptor would be indirect. Wild-type type II receptors tend to be distributed in a web-like manner throughout the cell, but do not have an appreciable apical localization (Fig 3B and 3D). However, this apical-to-basolateral distribution ratio is altered in the presence of mutant receptors, with a fraction of the mutant receptors now present at the apical surface. It is interesting to note that not all mutant receptors are apically localized. Since signaling complexes exist as heterotetrameric complexes, the composition of the complex might influence its trafficking route–a signaling complex made up of all mutant receptors might be apically localized while a complex containing a wild-type and a mutant receptor might be basolateral or ‘wild-type’ in its localization pattern.

### Altered trafficking of the MFS-like TGFβ type II receptor might localize it to areas with higher concentrations of ligand

The TGFβ signaling complex is tetrameric in nature [60], and altered trafficking of a single receptor type might lead the entire complex to mis-localize to a location that affects signaling strength. This is one way to envision how trafficking might affect signaling, although other possibilities exist. Spatial regulation of ligands and receptors is common in the TGFβ family [60]. In humans, TGFβ receptors are directed to basolateral membranes while TGFβ ligands are secreted at the apical surface of polarized cells [61]. Loss of the C-terminus LTA motif results in apically localized type II receptors, and apically targeted signaling complexes were fully functional as assayed by Smad3 phosphorylation [62], suggesting that all requirements for signaling exist at the apical surface. Because our nematode models of MFS-like mutations alter trafficking patterns, it is possible that the misdirected receptor complex is presented with a higher concentration of ligand at the apical surface. Receptor trafficking dynamics might also be altered at the apical surface such that the complex is retained for a longer time, allowing more signaling to occur before internalization and separation prior to recycling. Because our images are snapshots, it is not possible for us to identify whether receptor dynamics are altered at the surface. Does the LTA motif first direct an apical transit before basolateral sorting? If so, this would suggest that alterations to the motif might affect ‘transit time’ through the apical boundary before final delivery to the basolateral surface.

Although the LTA motif is known to direct trafficking, the molecules that interact with it are currently unknown. We have previously shown that the *C*. *elegans* type I receptor requires the retromer for recycling back to the surface while the type II receptor appears to require the small GTPase *arf-6*, whose loss leads to intra-endosomal accumulation of the type II receptor DAF-4 [52]. However, loss of the retromer leads to a redistribution from basolateral to a combination of basolateral and apical localization of the type II receptor in MD-1 cells [63], suggesting that receptors that were basolaterally localized have now been resorted to the apical membrane. Furthermore, loss of the retromer also reduced recycling by 50–60% in MD-1 cells [63]. The type I receptor requires the retromer for recycling, and perhaps this interaction can only take place in the context of the type II receptor LTA-motif-retromer interaction [63]. The presence of MFS-like mutations might interfere with this type I-type II-retromer complex and alter separation and recycling dynamics, thereby transiently increasing Smad phosphorylation. The inability of the type I receptor to then interact with the retromer would lead to its sorting into the lysosome for degradation, as we have previously shown. This is consistent with our data showing that the levels of the wild-type type I receptor are significantly reduced in the presence of the MFS-like mutant receptors (Fig 4A). Until new data are collected, other molecular scenarios may also be envisioned—MFS-mutations may disrupt a type II receptor-retromer interaction and lead to a redistribution of signaling complexes to the apical surface. Interestingly, many of these same mutations are also present in many cancer cell lines and tumor specimens (Fig 1A). It seems likely that some of these mutations also affect trafficking of the receptors.

Another recent study showed that in MFS patients, the TGFβ regulator SARA (Smad anchor for Receptor Activation) and SMAD2 is enriched within membrane fractions and leads to increased receptor interaction [64]. Importantly, the interaction of these mediators was significantly higher with the type II receptor. They further show that the TGFβ ligand has increased colocalization with SARA and EEA1 (an early endosome marker) which might lead to increased signaling. It would be very interesting to know whether the type II receptors in these patients contain mutations within their LTA domains.

Our *C*. *elegans* models of MFS-like syndromes offer a novel paradigm of MFS-like syndromes connecting receptor trafficking to disease. The mutations we have examined may provide possible therapeutic avenues; importantly some of these mutations are also found in cancers. If mutations are linked to trafficking defects in MFS-like syndromes then perhaps they share similar etiologies in other TGFβ receptor-induced cancers.

## Materials and methods

### Worm strains

All *C*. *elegans* strains were derived originally from the wild-type Bristol strain N2, and all strains were grown at 20°C on standard nematode growth media plates seeded with OP50 *E*. *coli*. Worm cultures, genetic crosses, and other *C*. *elegans* husbandry were performed according to standard protocols. RNAi was performed using the feeding method [65]. Feeding constructs were from the Ahringer library [66], and empty vector, L4440, was used as a control. For experiments, larval stage L4 animals were used, unless otherwise stated. The *daf-4(e1364)* mutant strain CB1364 was obtained from the CGC, and maintained at 15°C. For a list of strains, see S1 Table.

### Generation of plasmid constructs and transgenic animals

Intestinal expression was achieved through the intestinal-specific promoter, *vha-6*, using the following construct: *vha-6*p::*daf-4*::*gfp*::*unc-54 3’UTR* obtained from pRG78 [24]. The SL2 trans-splicing sequence was obtained from the Hobert lab and the *SV40*::*tdTomato*::*NLS* backbone from pCFJ1208 (pCFJ1208 was a gift from Erik Jorgensen—Addgene plasmid # 44490). The backbone plasmid pCFJ150 (contains unc-119(+), a rescue gene) was digested with SpeI and AflII. The four fragments were assembled using the NEB Hifi DNA Assembly Mastermix (catalog # E2621S) to generate the expression plasmid pJL22.

Hypodermal expression constructs: The hypodermal specific promoter, *elt-3* was amplified from pRG63 [52], *daf-4*::*gfp*::*SL2*::*SV40*::*tdTomato*::*NLS*::*unc54 3’UTR* from pJL22 and the vector backbone was prepared by digestion of pCFJ150 with SpeI and AflII. A three-fragment assembly was performed using the NEB Hifi DNA Assembly Mastermix (catalog # E2621S) to generate the final expression vector.

Selected MFS/MFS-like mutations were introduced into the *daf-4* receptor using the Q5 Site-directed mutagenesis kit from NEB (Catalog # E0554S) and were codon optimized for *C*. *elegans* based on the optimal codon usage [*C*. *elegans* II. 2nd edition, Table I).

All low-copy stably transformed strains were obtained through micro-particle bombardment. The protocol used was previously described in [67], well as the PDS-1000 / He and Hepta Systems from Bio-Rad. Macro particles carrier disk (catalog # 1652335), 2,000 psi Rupture Disks (catalog #1652333), 1.0 μm Gold Microcarriers (catalog #1652263) and Hepta Stopping Screens (catalog #1652226) were purchased from Bio-Rad. All microparticle bombardment was conducted in the *unc-119(ed3)* mutant background.

For all *pvha-6* and *pelt-3* constructs, 20 μg of DNA were used for microparticle bombardment.

All the transformed worm strains were outcrossed to N2 at least four times.

### Western blotting and quantification

Animals were synchronized by alkaline bleaching and arrested at L1 stage on unseeded NGM plates overnight. Arrested L1s were transferred to seeded plates and grown on standard NGM plates until L4 stage at 20°C. Fifty synchronized L4 stage worms for each strain were placed in 15 μl M9 buffer and 15 μl NextGel protein sample loading buffer (4x) (VWR, catalog # M260-5.0 ML) was added, flash frozen in liquid nitrogen and stored at -80°C until used for western blotting. Samples were boiled for 5 mins, then centrifuged at 13,000 rpm for 1 min. Samples were then loaded onto 10% polyacrylamide gels (NEXT GEL, Amresco, catalog # M256-500MLSG). Samples were run at 100V for 60–90 min. Transfer was performed using the Bio-Rad Trans-Blot Turbo Transfer System onto nitrocellulose membranes. Membranes were probed with anti-GFP from Roche (catalog # 11814460001), polyclonal goat anti-tdTomato from SCIGEN (catalog # AB8181-200), anti-actin and visualized using the Li-Cor Infrared imaging system. Quantification of band intensity was measured using Fiji Software and statistical comparisons were made using one-way ANOVA.

### Confocal imaging and quantification

Live worms were mounted on 2% (wt / vol) agarose pads with 0.1% tetramisole (MP Biomedicals, catalog # 0215211910) in M9 buffer. All confocal imaging was performed on the the Leica SP5 TCS confocal microscope using a lambda-scanning approach to minimize autofluorescence from the intestine as has been previously used in [52]. A minimum of six animals were imaged for each condition with a minimum of three biological replicates. Three randomly selected regions per animal were analyzed, using circular regions of defined area. Quantification of fluorescence intensities was performed. The average total intensity was calculated. Imaging quantification was performed using the open-source Fiji Software [68] on the third or fourth anterior pair of cells for the measurement of cytoplasmic GFP (cell membrane not included) and nuclear tdTomato. For the measurements of apical vs basolateral changes, the ‘segmented line’ tool was used to trace the respective surfaces and total GFP fluorescence intensities were calculated as above.

### Body length measurements and quantification

Whole body length measurement was performed by imaging on a standard epifluorescence microscope with the 5x objective. A minimum of thirty L4 worms per condition were imaged for three independent biological replicates. The individual values from all three trials were combined and presented as a combined graph with individual values to show the variation across trials. At least 30 animals per strain per trial were measured. Whole worm body lengths from head to tail along the axis were measured using the line tool in Fiji Software. Body lengths of mutants were compared to wild-type N2 control (Fig 2A) or to *daf-4(e1364)* (Fig 2B) and statistical differences were computed using a One-way ANOVA with multiple comparisons using Dunnett’s corrections.

### Structural modeling

The TGFβRII structural model was generated with the Swiss-model online program (https://swissmodel.expasy.org/) using template 5e8v.1.A for the TGFβ type II receptor). Representation of specific amino acids in the structural model is depicted by using the Pymol software. Minor conformational conflicts by disease-associated point mutations are shown by Pymol software [42].

## Supporting information

S1 Fig **A**. The LTA motif (highlighted in magenta onto the X-ray crystal structure of the kinase domain) is exposed to the environment, ripe for interaction with other proteins. Modeling of the MFS-like mutations suggest changes that might not impact the function of the kinase domain, but rather alter the interactions with other proteins. **B**. MFS-like substitutions are expected to lead to stearic hindrances as modeled by PyMol.
(TIF)

S2 Fig Transgenic expression of the daf-4::GFP (wild-type or MFS-mutant) was normalized by the operonic expression of a tdTomato transgene. As observed, there is no statistical difference between the tdTomato intensity amongst the strains showing that the transgenes are expressed at similar levels between the strains. Thus, the variation in body size can be explained by the intrinsic property of the various MFS-mutations.
(TIF)

S3 Fig *RNAi* mediated knockdown of *dpy-23* does not alter the levels of either wild-type or mutant DAF-4 receptors. Note: For mutant daf-4 constructs, images have been overexposed digitally to clearly observe differences, if any.
(TIF)

S4 Fig *RNAi* mediated knockdown of *cup-5* to reduce lysosomal function does not increase levels of either wild-type or mutant DAF-4 receptors suggesting that the decreased levels of MFS-like mutant DAF-4 receptors are not a result of degradation within the lysosome. Note: For mutant daf-4 constructs, images have been overexposed digitally to clearly observe differences, if any.
(TIF)

S5 Fig Transgenic expression of the untagged daf-4 (wild-type or MFS-mutant) was normalized by the operonic expression of a tdTomato transgene. As observed, there is no statistical difference between the tdTomato intensity amongst the strains showing that the transgenes are expressed at similar levels between the strains.
(TIF)

S1 Table List of strains used in the study. (DOCX)
