# Supplementary figures and images for: Human Marfan and Marfan-like Syndrome associated mutations lead to altered trafficking of the Type II TGFβ receptor in Caenorhabditis elegans
Source: PLoS One. 2019 May 9;14(5):e0216628. doi: 10.1371/journal.pone.0216628 (PMC6508650; doi:10.1371/journal.pone.0216628)

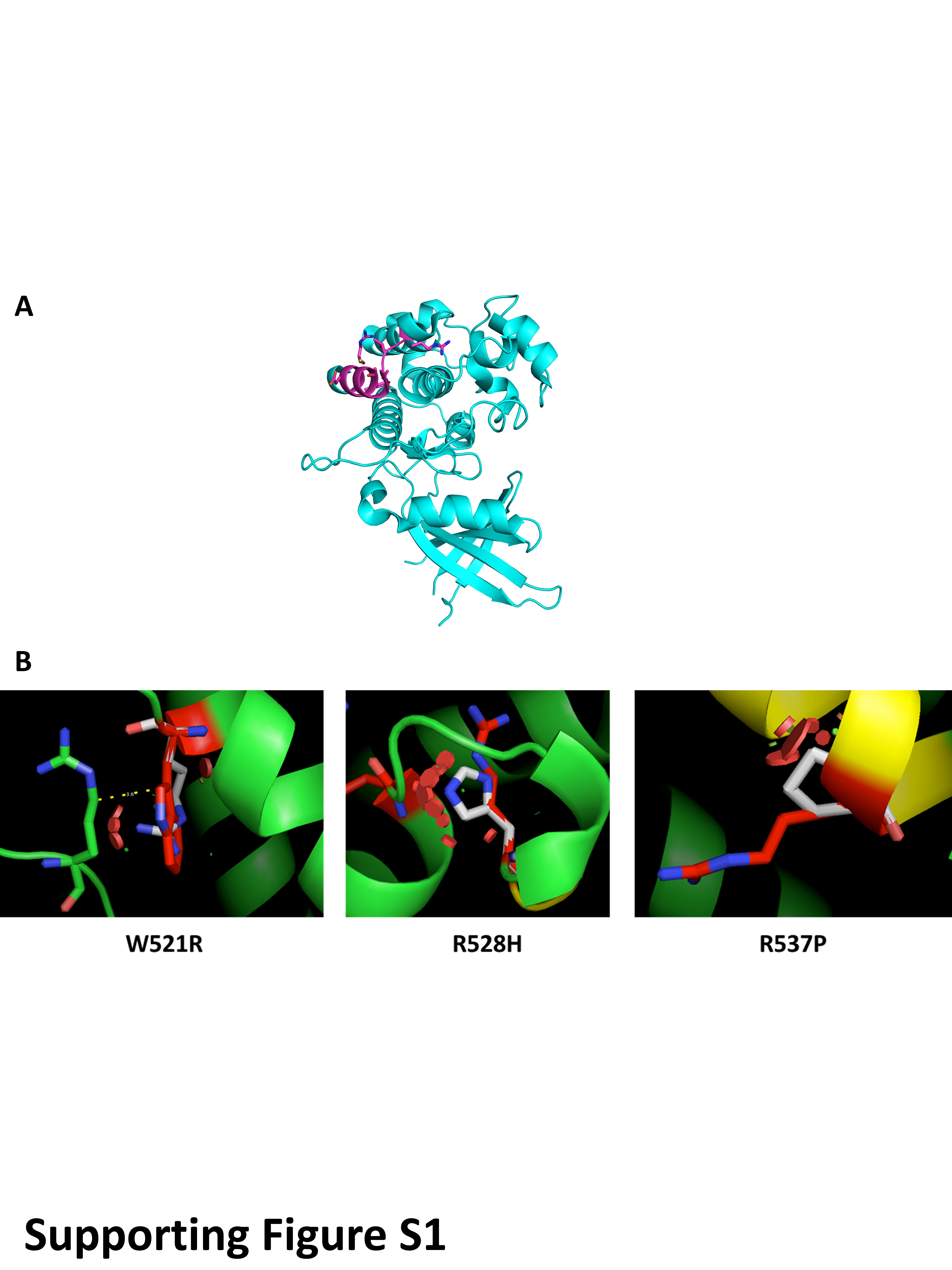

Supplement: S1 Fig — A. The LTA motif (highlighted in magenta onto the X-ray crystal structure of the kinase domain) is exposed to the environment, ripe for interaction with other proteins. Modeling of the MFS-like mutations suggest changes that might not impact the function of the kinase domain, but rather alter the interactions with other proteins. B. MFS-like substitutions are expected to lead to stearic hindrances as modeled by PyMol. (TIF) [file pone.0216628.s001.TIF]

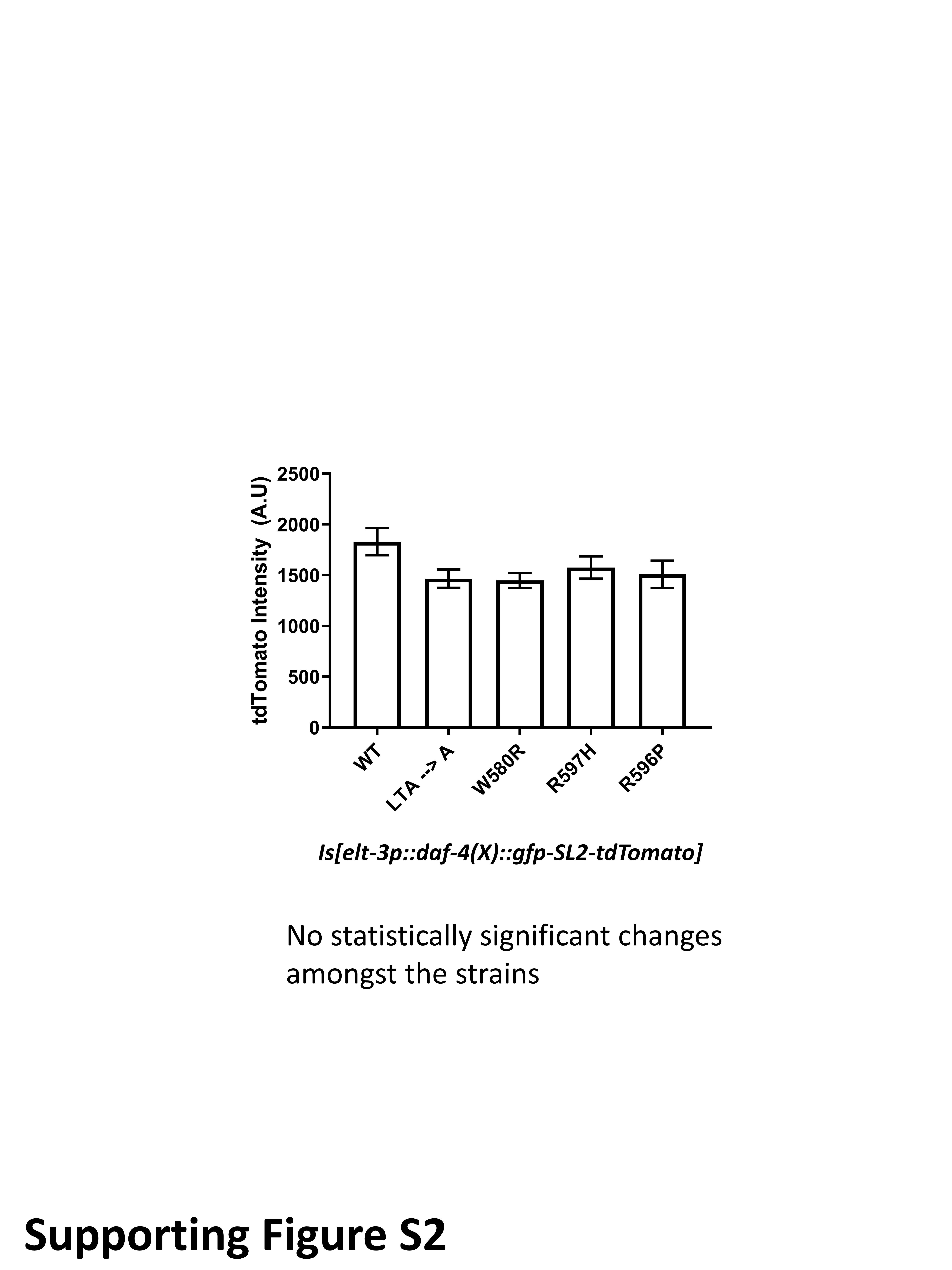

Supplement: S2 Fig — As observed, there is no statistical difference between the tdTomato intensity amongst the strains showing that the transgenes are expressed at similar levels between the strains. Thus, the variation in body size can be explained by the intrinsic property of the various MFS-mutations. (TIF) [file pone.0216628.s002.TIF]

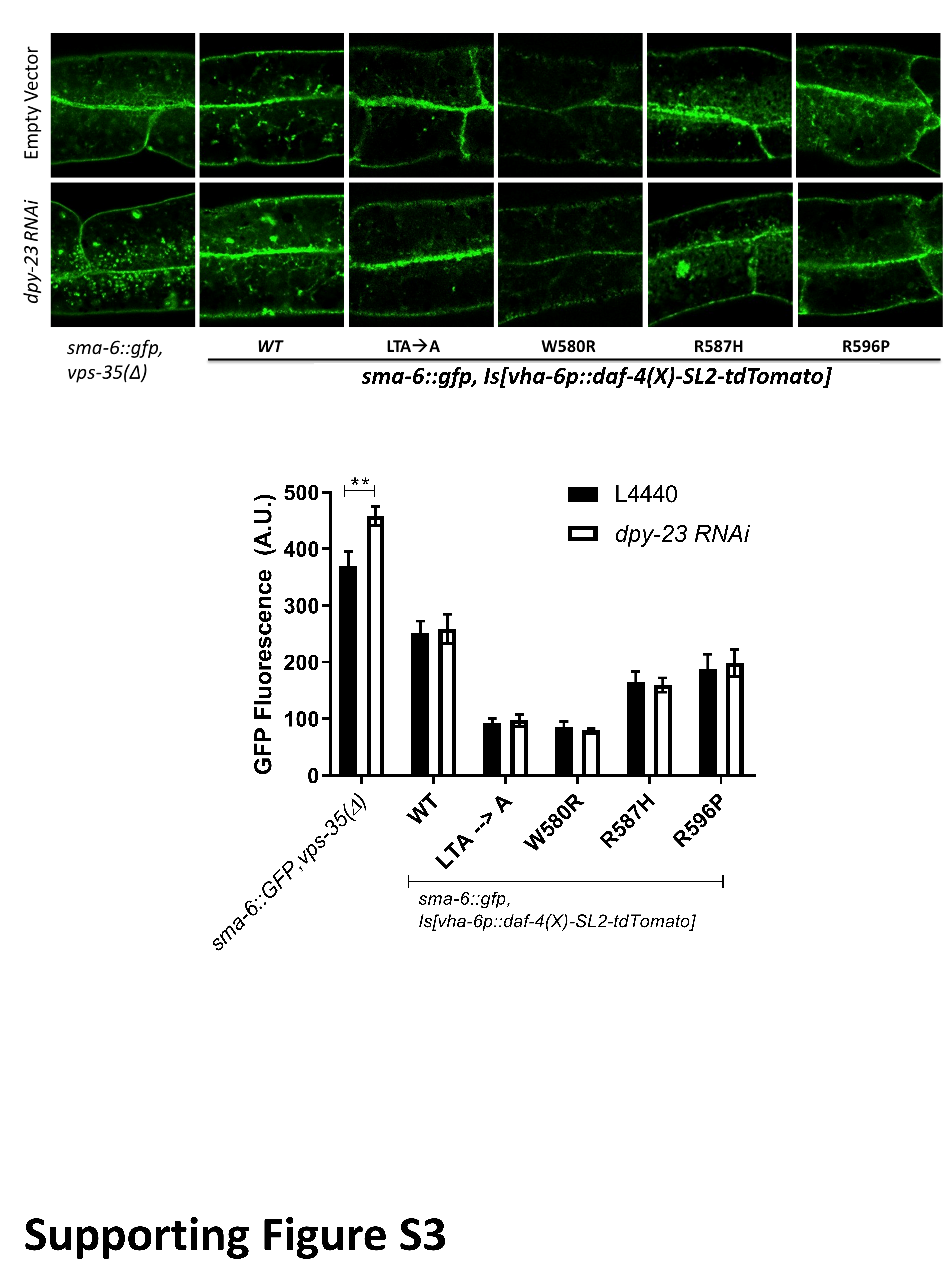

Supplement: S3 Fig — Note: For mutant daf-4 constructs, images have been overexposed digitally to clearly observe differences, if any. (TIF) [file pone.0216628.s003.TIF]

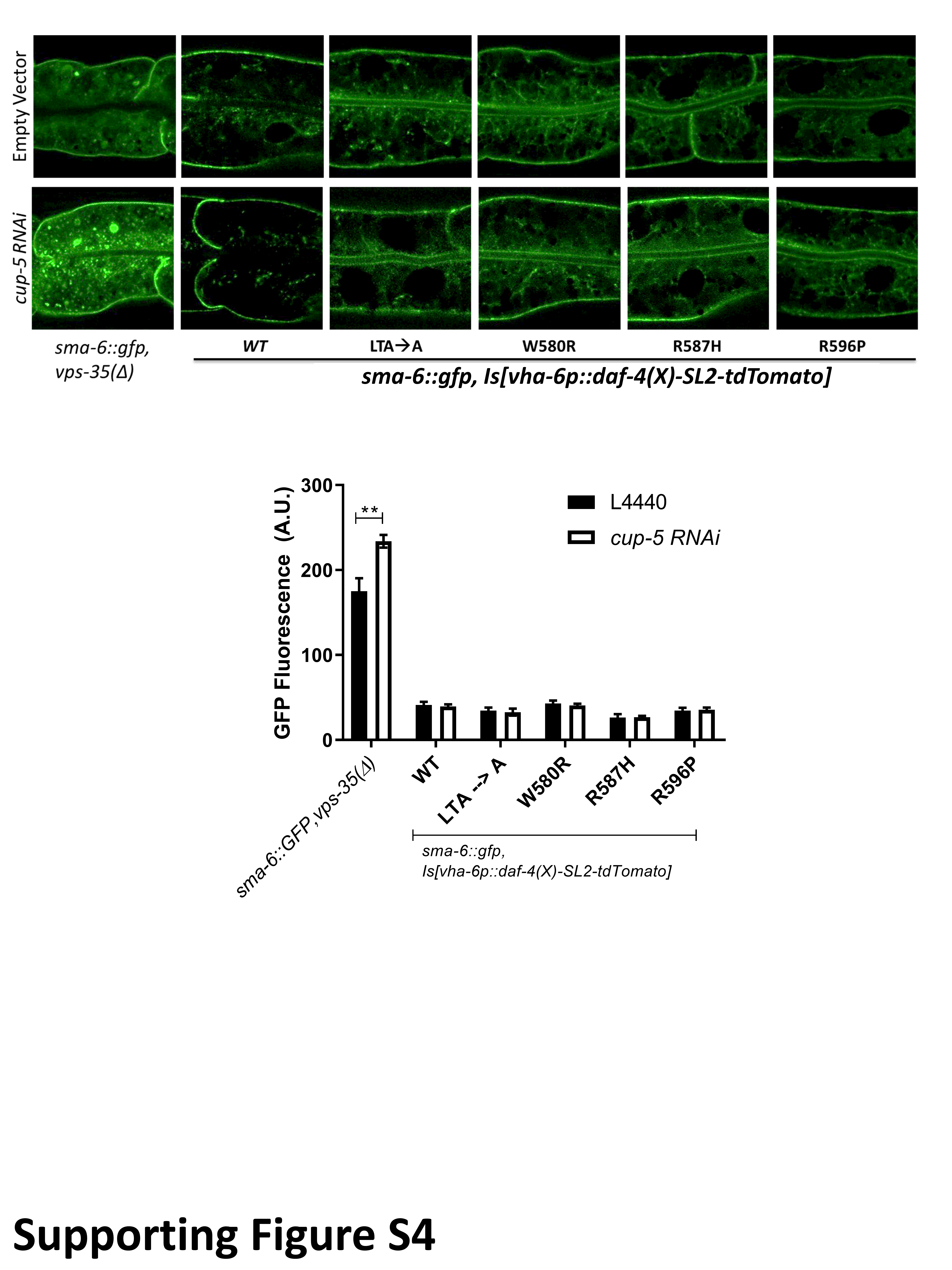

Supplement: S4 Fig — Note: For mutant daf-4 constructs, images have been overexposed digitally to clearly observe differences, if any. (TIF) [file pone.0216628.s004.TIF]

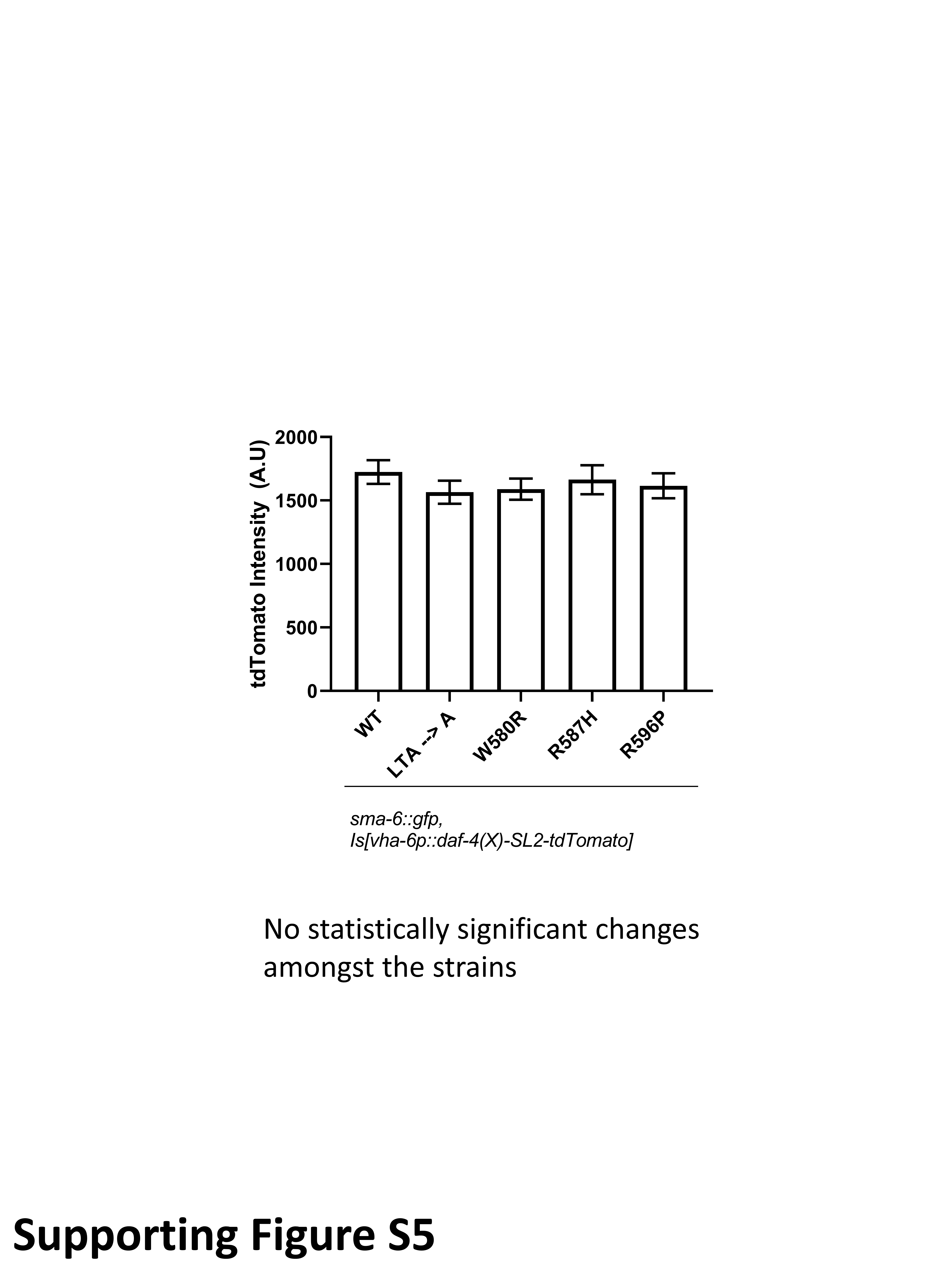

Supplement: S5 Fig — As observed, there is no statistical difference between the tdTomato intensity amongst the strains showing that the transgenes are expressed at similar levels between the strains. (TIF) [file pone.0216628.s005.TIF]
