# Supplementary material for: Human Marfan and Marfan-like Syndrome associated mutations lead to altered trafficking of the Type II TGFβ receptor in Caenorhabditis elegans
Source: PLoS One. 2019 May 9;14(5):e0216628. doi: 10.1371/journal.pone.0216628 (PMC6508650; doi:10.1371/journal.pone.0216628)
Supplement: S1 Table — (DOCX) [file pone.0216628.s006.docx]

**S1 Table: List of strains used in the study**

| **STRAIN NAME** | **Genotype** |
| --- | --- |
| LT1009 | *vha-6p::daf-4(WT)-gfp-sl2-sv40-NLS-tdTomato-NLS-unc-54(3'UTR)* |
| LT1010 | *vha-6p::daf-4(W580R)-gfp-sl2-sv40-NLS-tdTomato-NLS-unc-54(3'UTR)* |
| LT1011 | *vha-6p::daf-4(LTA🡪As)-gfp-sl2-sv40-NLS-tdTomato-NLS-unc-54(3'UTR)* |
| LT1012 | *vha-6p::daf-4(R587H)-gfp-sl2-sv40-NLS-tdTomato-NLS-unc-54(3'UTR)* |
| LT1013 | *vha-6p::daf-4(R596P)-gfp-sl2-sv40-NLS-tdTomato-NLS-unc-54(3'UTR)* |
| LT1014 | *vha-6p::daf-4(WT)-sl2-sv40-NLS-tdTomato-NLS-unc-54(3'UTR)* |
| LT1015 | *vha-6p::daf-4(W580R)-sl2-sv40-NLS-tdTomato-NLS-unc-54(3'UTR)* |
| LT1016 | *vha-6p::daf-4(LTA🡪As)-sl2-sv40-NLS-tdTomato-NLS-unc-54(3'UTR)* |
| LT1017 | *vha-6p::daf-4(R587H)-sl2-sv40-NLS-tdTomato-NLS-unc-54(3'UTR)* |
| LT1018 | *vha-6p::daf-4(R596P)-sl2-sv40-NLS-tdTomato-NLS-unc-54(3'UTR)* |
| LT1041 | *vha-6p::sma-6(WT)-gfp;*  *vha-6p::daf-4(WT)-sl2-sv40-NLS-tdTomato-NLS-unc-54(3'UTR)* |
| LT1042 | *vha-6p::sma-6(WT)-gfp;*  *vha-6p::daf-4(W580R)-sl2-sv40-NLS-tdTomato-NLS-unc-54(3'UTR)* |
| LT1043 | *vha-6p::sma-6(WT)-gfp;*  *vha-6p::daf-4(LTA🡪As)-sl2-sv40-NLS-tdTomato-NLS-unc-54(3'UTR)* |
| LT1044 | *vha-6p::sma-6(WT)-gfp;*  *vha-6p::daf4(R587H)-sl2-sv40-NLS-tdTomato-NLS-unc-54(3'UTR)* |
| LT1045 | *vha-6p::sma-6(WT)-gfp;*  *vha-6p::daf4(R596P)-sl2-sv40-NLS-tdTomato-NLS-unc-54(3'UTR)* |
| LT1019 | *elt-3p::daf-4(WT)-gfp-sl2-sv40-NLS-tdTomato-NLS-unc-54(3'UTR)* |
| LT1020 | *elt-3p::daf-4(W580R)-gfp-sl2-sv40-NLS-tdTomato-NLS-un-c54(3'UTR)* |
| LT1021 | *elt-3p::daf-4(LTA🡪As)-gfp-sl2-sv40-NLS-tdTomato-NLS-unc-54(3'UTR)* |
| LT1022 | *elt-3p::daf-4(R587H)-gfp-sl2-sv40-NLS-tdTomato-NLS-unc-54(3'UTR)* |
| LT1023 | *elt-3p::daf-4(R596P)-gfp-sl2-sv40-NLS-tdTomato-NLS-unc-54(3'UTR)* |
| LT1046 | *elt-3p::daf-4(K388R)-gfp-sl2-sv40-NLS-tdTomato-NLS-unc-54-3’UTR* |
| LT831 | *vha-6p::sma-6(WT)-gfp(RT2496); vps-35 (hu35)* |
| CB1364 | *daf-4(e1364) III* |
